# Pet ownership, pet-related characteristics, and sleep quality among older adults in China: a nationwide cross-sectional study

**DOI:** 10.3389/fvets.2025.1721332

**Published:** 2026-02-02

**Authors:** Yixin Tang, Nini Zhu, Mengqi Jin, Zhangqing Ren, Jianliang Lu, Lili Xu, Lijun Xie, Jianjiang Pan

**Affiliations:** 1 Department of General Practice, Sir Run Run Shaw Hospital, School of Medicine, Zhejiang University, Hangzhou, China; 2 Department of General Practice, Affiliated Lianyungang Clinical College of Nantong University & The Second People's Hospital of Lianyungang, Lianyungang, China; 3 Department of General Practice, Shaoxing People's Hospital, Shaoxing, China; 4 Department of Emergency, Ninth People's Hospital of Zhengzhou (Geriatric Hospital of Zhengzhou), Zhengzhou, China; 5 Department of General Practice, First Affiliated Hospital of Shihezi University, Shihezi, China; 6 Department of General Practice, The Second Affiliated Hospital of Xi'an Medical University, Xi'an, China

**Keywords:** China, cross-sectional study, older adults, pet ownership, sleep quality

## Abstract

**Introduction:**

As population aging accelerates globally, sleep disorders among older adults have become a significant public health concern. While pet ownership is increasingly recognized for its psychosocial benefits, its specific impact on sleep quality remains inconclusive. This study aimed to investigate the associations between pet ownership, diverse pet-related characteristics, and sleep quality among the elderly in China.

**Methods:**

A nationwide cross-sectional study was conducted involving 1,434 Chinese participants aged 60 and above (mean age: 71.0 ± 7.0 years). Sleep quality was assessed using the Athens Insomnia Scale (AIS). Multivariate logistic regression models were employed to estimate the adjusted odds ratios (ORs) and 95% confidence intervals (CIs) for insomnia risk, adjusting for sociodemographic factors, health status, and lifestyle habits.

**Results:**

Pet ownership was significantly associated with a reduced risk of insomnia (adjusted OR = 0.75, 95% CI: 0.59–0.96). Specifically, dog ownership (OR = 0.63, 95% CI: 0.40–0.98) and owning healthy pets (OR = 0.41, 95% CI: 0.23–0.70) were protective factors. Conversely, rabbit ownership (OR = 1.94, 95% CI: 1.04–3.59), ownership duration of less than one year, and daily interaction exceeding two hours were associated with an increased risk of poor sleep quality. Frequent dog walking was also found to be beneficial for sleep health.

**Discussion:**

Our findings demonstrate that the pet-sleep relationship in older adults is complex and contingent on specific ownership contexts, suggesting that targeted pet companionship may serve as a potential intervention for sleep health in aging populations.

## 1 Introduction

Population aging has emerged as a major challenge in global public health. By 2023, the global population aged 60 and above reached approximately 1.1 billion, projected to increase to 2.1 billion by 2050, accounting for 16% of the global population (1). Sleep disorders are particularly common in older adults, with prevalence estimates ranging from 30% to 70% (2). Poor sleep quality is linked not only daytime fatigue and impaired concentration but also to long-term consequences such as cognitive decline, cardiovascular disease, and reduced quality of life (3). The additional healthcare burden imposed by insomnia in older adults is substantial, placing significant strain on both individuals and society (4). Improving sleep quality is therefore recognized as a cornerstone of healthy aging, and finding feasible intervention strategies to support better sleep has become a public health priority (4–6).

Pets are now a familiar part of daily life, with more than half of households worldwide owning one (7). Previous studies indicate that pet companionship can alleviate loneliness, provide emotional support, and promote physical and mental health by increasing physical activity, all of which can benefit sleep (8). For instance, dog owners may experience shorter sleep latency and improved sleep continuity due to increased daily physical activity from walking their pets (9). However, pet ownership may also carry adverse effects, such as sleep disruption caused by nocturnal pet activity or psychological burdens from increased caregiving responsibilities (10). Current evidence on the association between pet ownership and sleep remains inconsistent.

Despite growing interest in the health benefits of pet ownership, current evidence regarding its association with sleep quality remains significantly limited. Many studies reduce pet ownership to a binary measure “owning a pet or not” without considering important contextual factors such as duration of ownership, level of daily interaction, pet health, or the financial resources required to care for a pet (10–12). These dimensions may critically shape whether pet ownership has beneficial, neutral, or even adverse effects on sleep. Few studies have compared different types of pets, even though dogs, cats, birds, and small mammals vary substantially in their behavioral patterns, care requirements, and the kinds of interactions they foster with humans (13). Such differences may translate into distinct effects on sleep quality. Evidence is particularly scarce among older adults, who face unique challenges such as higher rates of social isolation, reduced physical activity, and increased vulnerability to sleep disorders (11, 14). Yet, the specific mechanisms through which pet companionship functions within this particular population remain unclear. These gaps are even more pronounced in the Chinese context, and research mainly focuses on young people (15). With rapid population aging and the proliferation of “empty-nest” households, companion animals are assuming an increasingly vital role in the lives of older adults (16). However, domestic studies have rarely investigated sleep as a primary health outcome. As a result, the role of pet companionship in sleep health among Chinese older adults remains largely unexplored.

To bridge these gaps, this study utilizes a nationwide, multi-center sample to comprehensively investigate the association between pet ownership behaviors and sleep quality among Chinese adults aged 60 years and older. Moving beyond the assessment of simple ownership status, we integrated multidimensional characteristics including pet type, duration of ownership, daily interaction, pet health status, and related expenses. By doing so, we aim to unravel the complex associations between these factors and sleep quality, thereby providing localized empirical evidence for understanding the potential role of pets in the healthy aging process of Chinese older adults.

## 2 Methods

### 2.1 Study design and participants

This cross-sectional, multicenter survey was conducted between August 2024 and January 2025 to examine the relationship between pet ownership and sleep quality among older adults in China. Data were collected using a standardized, structured questionnaire administered through face-to-face interviews.

Inclusion and Exclusion Criteria: Participants were eligible if they were aged 60 years or older, had sufficient cognitive and communication ability to understand and complete the questionnaire independently or with interviewer assistance, and provided written informed consent. Individuals were excluded if they had clinically diagnosed severe cognitive impairment (e.g., dementia) or serious physical or psychiatric conditions (e.g., terminal cancer, unstable heart failure, major depressive episode, or schizophrenia) that prevented participation.

### 2.2 Sample size calculation

The sample size of the present study was determined based on previous studies reporting an insomnia prevalence of approximately 30–40% among older adults in China (17). Given a power of 95% and a two-sided significance level of 0.05, this study required minimum sample size of 600. To ensure adequate power for subgroup analyses involving different pet-related characteristics and to account for a potential non-response rate of 20%, we increased the target sample to 1,440 participants.

### 2.3 Sampling strategy

A multistage mixed sampling strategy was employed to obtain a representative sample of older adults from diverse geographical regions across China. In the first stage, five provinces were purposively selected to capture geographic, economic, and cultural diversity: Zhejiang and Jiangsu (eastern China, economically developed), Henan (central China, moderate development), and Shaanxi and Xinjiang (western China, less developed and more ethnically diverse). This approach ensured coverage of both urban and rural populations across different socioeconomic contexts. In the second stage, three to four community health service centers were identified within each province. Centers were chosen based on their capacity to collaborate in research, accessibility to local residents, and ability to provide a stable sampling frame of older adults regularly attending for routine health services. In the third stage, quota sampling was used to recruit 80~100 participants per site. To minimize imbalance between exposure groups, an alternating recruitment procedure was adopted: after enrolling one eligible pet owner, the next eligible non-owner was recruited, and vice versa. This ensured approximately equal numbers of pet owners and non-owners within each site, facilitating direct comparison. All participants provided informed consent to participate voluntarily in the survey, and the study was approved by the Ethics Committee of Zhejiang University School of Public Health (approval no. 202406-8).

### 2.4 Study variables

#### 2.4.1 Sleep quality

Sleep quality was assessed using the Athens Insomnia Scale (AIS) (18). This scale consists of eight items, each rated on a 4-point Likert scale ranging from 0 to 3. The total score is calculated by summing all items, with higher scores indicating more severe insomnia symptoms. A cutoff score greater than 6 was considered indicative of insomnia. The AIS demonstrated a Cronbach's α coefficient of 0.91 in this study, indicating high internal consistency.

#### 2.4.2 Pet ownership and characteristics

The exposure variable in this study is pet ownership behavior, with relevant data collected using a structured, self-developed questionnaire. Pet ownership status was first assessed by asking participants whether they currently kept any household pets. Based on their responses, individuals were classified as pet owners or non-owners. For pet owners, additional information on pet-related characteristics was obtained.

Pet type was measured through four separate yes/no questions asking whether participants owned a dog, cat, bird, or rabbit. These items were not mutually exclusive, allowing respondents to report multiple pet types. For analysis, each pet type was coded as an independent binary variable.

Additional pet-related characteristics were measured using standardized categories. Duration of ownership was classified as < 1 year, 1–3 years, or >3 years. Daily interaction time referred to the average amount of time spent with the pet per day (< 1 h, 1–2 h, >2 h). Pet health status was assessed through a three-level self-rating (poor, fair, good), based on the participant's perception. Annual pet-related expenses included spending on food, veterinary care, grooming, and supplies (< 1,000 RMB; 1,000–2,999 RMB; ≥3,000 RMB).

The questionnaire was developed based on a review of prior epidemiological research on pet ownership and was reviewed by experts in public health and gerontology to ensure content relevance for older adults (19). Given that these items reflect concrete behaviors or factual attributes rather than latent constructs, psychometric measures such as internal consistency were not applicable. To promote measurement consistency, all interviewers received centralized training and followed a standardized script when administering the questionnaire.

#### 2.4.3 Covariates

Sociodemographic characteristics included age, gender, ethnicity, marital status, region, housing situation, educational attainment, employment status, monthly income, and smoking and drinking habits.

### 2.5 Statistical analysis

All analyses were conducted using STATA version 16.0, with a two-sided significance level of *p* < 0.05. Missing data accounted for less than 1% of all observations. Given the negligible proportion, complete-case analysis was applied in all regression models. These cases were retained in the dataset and coded as “.” in STATA. Descriptive statistics were first generated to summarize sociodemographic characteristics, pet ownership, and sleep outcomes. Categorical variables were expressed as counts and percentages, while continuous variables were expressed as means with standard deviations or medians with interquartile ranges, depending on distribution. Group differences were tested using chi-square tests for categorical variables and *t*-tests or non-parametric tests for continuous variables, as appropriate. Binary logistic regression was then used to estimate the association between pet ownership (yes/no) and insomnia, defined as AIS > 6. A multivariate logistic regression model assessed the association between pet ownership status and insomnia, controlling for potential confounders including age, gender, marital status, education level, housing situation, income level, chronic disease history, and smoking/drinking habits. Furthermore, among pet owners, multivariable logistic regression was applied to examine associations between specific pet-related characteristics (type of pet, duration of ownership, daily interaction time, pet health status, and annual expenses) and insomnia.

## 3 Results

A total of 1,507 questionnaires were distributed, with 1,434 valid responses collected, yielding a response rate of 95.2%. The average age of participants was 71.0 ± 7.0 years, with women accounting for 53.0%. According to the Athens Insomnia Scale (AIS), 414 participants (28.9%) were identified as having insomnia.

### 3.1 Basic characteristics of the research subjects

Table 1 presents the demographic and sociodemographic characteristics of the subjects, stratified by sleep quality. Compared with the non-insomnia group, the insomnia group had a higher proportion of females (61.2% vs. 49.7%, *p* < 0.01), a higher proportion of widowed individuals (20.7% vs. 12.7%, *p* < 0.01), a higher proportion residing in the central region (23.9% vs. 10.8%, *p* < 0.01), and a higher proportion of urban residents (60.2% vs. 50.3%, *p* < 0.01). Regarding educational attainment, the insomnia group had a higher proportion with primary school education or below and a lower proportion with secondary school education or above. Regarding lifestyle, both smoking and alcohol consumption rates were lower in the insomnia group than in the non-insomnia group. Economically, a lower proportion of the insomnia group had monthly incomes ≥5,000 yuan (16.1% vs. 27.0%, *p* < 0.01). Pet ownership was also less common in the insomnia group (44.3% vs. 52.3%, *p* < 0.01).

**Table 1:** Characteristics of participants stratified by sleep quality (*n* = 1,434).

**Demographic characteristics**	**Total (*n* = 1,432 %)**	**No insomnia (*n* = 1,018 %)**	**Insomnia (*n* = 414 %)**	***T***	**χ^2^**	***p***
Age (years)	71.0 ± 7.0	70.7 ± 6.8	71.7 ± 7.4	−2.1		< 0.05
BMI (kg/m^2^)	24.0 ± 3.7	24.1 ± 3.6	23.9 ± 3.8	0.9		0.37
**Gender**					15.8	< 0.01
Male	674 (47.0)	513 (50.3)	161 (38.8)			
Female	760 (53.0)	506 (49.7)	254 (61.2)			
**Ethnicity**					0.9	0.33
Han Chinese	1,422 (99.2)	1,012 (99.3)	410 (98.8)			
Other ethnic groups	12 (0.8)	7 (0.7)	5 (1.2)			
**Marital status**					15.8	< 0.01
Married	1,163 (81.1)	852 (83.6)	311 (74.5)			
Widowed	215 (15.0)	129 (12.7)	86 (20.7)			
Other	56 (3.9)	38 (3.7)	18 (4.3)			
**Region**					40.4	< 0.01
Western China	694 (48.4)	514 (50.4)	180 (43.4)			
Central China	209 (14.6)	110 (10.8)	99 (23.9)			
Eastern China	531 (37.0)	395 (38.8)	136 (32.7)			
**Residence**					11.6	0.01
Urban	763 (53.2)	513 (50.3)	250 (60.2)			
Rural	671 (46.8)	506 (49.7)	165 (39.8)			
**Residential status**					8.6	0.01
Living with partner	1,038 (72.4)	760 (74.6)	278 (67.0)			
Living alone	217 (15.5)	141 (13.9)	76 (18.3)			
Living with children or others	179 (12.5)	118 (11.5)	61 (14.7)			
**Education attainment**					6.6	0.08
Uneducated	237 (16.5)	157 (15.4)	80 (19.3)			
Primary school	460 (32.0)	318 (31.2)	142 (34.2)			
Middle or high school	615 (42.9)	451 (44.3)	164 (39.5)			
College or above	122 (8.5)	93 (9.1)	29 (7.0)			
**Employment status**					13.2	< 0.01
Retired	1,227 (85.6)	850 (83.4)	377 (90.8)			
Not retired	207 (14.4)	169 (16.6)	38 (9.2)			
**Smoke**					9.8	< 0.01
Yes	460 (32.1)	352 (34.5)	108 (26.0)			
No	974 (67.9)	667 (65.5)	307 (74.0)			
**Drinking**					14.2	< 0.01
Yes	475 (33.1)	368 (36.1)	107 (25.8)			
No	959 (66.9)	651 (63.9)	308 (74.2)			
**Monthly income (RMB yuan)**					25.7	< 0.01
< 1,000	220 (15.3)	161 (15.8)	59 (15.3)			
≥1,000	306 (21.3)	195 (19.1)	111 (26.8)			
≥3,000	457 (31.9)	309 (30.3)	148 (35.7)			
≥5,000	342 (23.8)	275 (27.0)	67 (16.1)			
≥10,000	109 (7.6)	79 (7.8)	30 (7.2)			
**Pet ownership**					7.5	< 0.01
Non-pet ownership	717 (50.0)	486 (47.7)	231 (55.7)			
Pet ownership	717 (50.0)	533 (52.3)	184 (44.3)			

### 3.2 Multivariate analysis of the entire sample

Table 2 shows the results of the multivariable logistic regression analysis for the entire sample. After controlling for demographic, socioeconomic, and lifestyle covariates, pet ownership was significantly associated with a reduced risk of insomnia (OR = 0.75, 95% CI: 0.59–0.96, *p* < 0.05). Other factors significantly associated with insomnia included: being widowed (OR = 1.65, 95% CI: 1.02–2.66, *p* < 0.05), residing in the central region (OR = 2.58, 95% CI: 1.77–3.76, *p* < 0.01), rural residence (OR = 0.49, 95% CI: 0.36–0.67, *p* < 0.01), higher education level (secondary/high school: OR = 0.55, 95% CI: 0.37–0.81; college and above: OR = 0.45, 95% CI: 0.24–0.82), and non-retired status (OR = 0.65, 95% CI: 0.44–0.98, *p* < 0.05).

**Table 2:** Results of binary logistic regression models by sleep quality.

**Demographic characteristics**	**OR (95%CI)**	***p***
Age (years)	1.00 (0.97, 1.01)	0.71
BMI (kg/m^2^)	1.00 (0.99, 1.00)	0.86
**Gender**		
Male	1.00	
Female	1.20 (0.88, 1.65)	0.25
**Ethnicity**		
Han Chinese	1.00	
Other ethnic groups	1.27 (0.35, 4.70)	0.71
**Marital status**		
Married	1.00	
Widowed	1.65 (1.02, 2.66)	< 0.05
Other	1.24 (0.61, 2.52)	0.55
**Region**		
Western China	1.00	
Central China	2.58 (1.77, 3.76)	< 0.01
Eastern China	0.91 (0.67, 1.23)	0.55
**Residence**		
Urban	1.00	
Rural	0.49 (0.36, 0.67)	< 0.01
**Residential status**		
Living with partner	1.00	
Living alone	1.07 (0.68, 1.70)	0.76
Living with children or others	0.86 (0.52, 1.37)	0.50
**Education attainment**		
Uneducated	1.00	
Primary school	0.91 (0.63, 1.30)	0.59
Middle or high school	0.55 (0.37, 0.81)	< 0.01
College or above	0.45 (0.24, 0.82)	< 0.01
**Employment status**		
Retired	1.00	
Not retired	0.65 (0.44, 0.98)	< 0.05
**Smoke**		
Yes	1.00	
No	0.99 (0.70, 1.42)	0.98
**Drinking**		
Yes	1.00	
No	1.29 (0.93, 1.79)	0.12
**Monthly income (RMB yuan)**		
< 1,000	1.00	
≥1,000	1.51 (1.00, 2.27)	< 0.05
≥3,000	1.19 (0.79, 1.80)	0.41
≥5,000	0.83 (0.51, 1.34)	0.44
≥10,000	1.46 (0.78, 2.73)	0.24
**Pet ownership**		
Non-pet ownership	1.00	
Pet ownership	0.75 (0.59, 0.96)	< 0.05

### 3.3 Segmentation analysis of pet owners

Among the 717 participants who owned pets, the prevalence of insomnia was 25.7%. Table 3 shows that certain pet ownership characteristics were associated with insomnia. The insomnia group had a higher proportion of pet ownership duration under 1 year (39.7% vs. 26.1%, *p* < 0.01), a higher proportion of pets in “poor” condition (27.2% vs. 12.4%, *p* < 0.01), a higher proportion spent less than ¥1,000 annually on pet care (71.7% vs. 62.5%, *p* < 0.05), a higher proportion walked their pets “rarely” (52.2% vs. 43.7%, *p* < 0.05), and a higher proportion owned rabbits (14.7% vs. 8.1%, *p* < 0.01).

**Table 3:** Characteristics of participants of pet ownership by sleep quality (*N* = 717).

**Demographic characteristics**	**Total (*n* = 717 %)**	**No insomnia (*n* = 533 %)**	**Insomnia (*n* = 184 %)**	**χ^2^**	***p***
**Pet ownership duration**				13.3	< 0.01
< 1 year	212 (29.6)	139 (26.1)	73 (39.7)		
1–3 years	182 (25.4)	137 (25.7)	45 (24.5)		
>3 years	323 (45.0)	257 (48.2)	67 (35.8)		
**Pet health status**				23.1	< 0.01
Poor	116 (16.2)	66 (12.4)	50 (27.2)		
Fair	307 (42.8)	244 (45.8)	63 (34.2)		
Good	294 (41.0)	223 (41.8)	71 (38.6)		
**Annual pet-related expenses (RMB yuan)**				6.2	< 0.05
< 1,000	465 (64.8)	333 (62.5)	132 (71.7)		
1,000–2,999	194 (27.1)	151 (28.3)	43 (23.4)		
≥3,000	58 (8.1)	49 (9.2)	9 (4.9)		
**Pet walking frequency**				7.9	< 0.05
Almost every day	201 (28.0)	164 (30.8)	37 (20.1)		
Several times a week	187 (26.1)	136 (25.5)	51 (27.7)		
Rarely	329 (45.9)	233 (43.7)	96 (52.2)		
**Daily interaction time with pets**				0.79	0.68
< 1 h	381 (53.1)	280 (52.5)	101 (54.9)		
1–2 h	161 (22.5)	124 (23.3)	37 (20.1)		
>2 h	175 (24.4)	129 (24.2)	46 (25.0)		
**Dog ownership**				14.8	< 0.01
No	230 (32.1)	150 (28.1)	80 (43.5)		
Yes	487 (67.9)	383 (71.9)	104 (56.5)		
**Cat ownership**				1.65	0.20
No	447 (62.3)	325 (61.0)	122 (66.3)		
Yes	270 (37.7)	208 (39.0)	62 (33.7)		
**Bird ownership**				1.8	0.18
No	656 (91.5)	492 (92.3)	164 (89.1)		
Yes	61 (8.5)	41 (7.7)	20 (10.9)		
**Rabbit ownership**				6.8	< 0.01
No	647 (90.2)	490 (91.9)	157 (85.3)		
Yes	70 (9.8)	43 (8.1)	27 (14.7)		

### 3.4 Multivariate analysis of pet ownership characteristics and insomnia

Table 4 further presents the results of the multivariable logistic regression analysis examining the association between pet ownership characteristics and insomnia. After adjusting for covariates, pet health status was significantly associated with insomnia: Older adults with “good” pets had a lower risk of insomnia than those with “poor” pets (OR = 0.41, 95% CI: 0.23–0.70, *p* < 0.01). Older adults with lower pet walking frequency had a higher likelihood of insomnia. Compared to those who walked their pets “almost daily,” those who walked “several times a week” (OR = 1.79, 95% CI: 1.03–3.11, *p* < 0.05) and those who walked “rarely” (OR = 1.97, 95% CI: 1.13–3.42, *p* < 0.05) were associated with insomnia. Older adults spending over 2 h daily with pets had a higher likelihood of insomnia (OR = 2.25, 95% CI: 1.27–3.99, *p* < 0.01). Regarding pet type, dog ownership was associated with a reduced insomnia risk (OR = 0.63, 95% CI: 0.40–0.98, *p* < 0.05), while rabbit ownership was associated with an increased insomnia risk (OR = 1.94, 95% CI: 1.04–3.59, *p* < 0.05); No significant differences were observed for cat ownership (*p* = 0.34) or bird ownership (*p* = 0.06).

**Table 4:** Results of binary logistic regression models by sleep quality (*N* = 717).

**Demographic characteristics**	**OR (95%CI)**	***p***	**OR (95%CI)**	***p***	**OR (95%CI)**	***p***	**OR (95%CI)**	***p***
**Pet ownership duration**								
< 1 year	1.00		1.00		1.00		1.00	
1–3 years	0.85 (0.51, 1.40)	0.51	0.78 (0.47, 1.29)	0.33	0.84 (0.51, 1.40)	0.50	0.82 (0.50, 1.36)	0.44
>3years	0.71 (0.44, 1.13)	0.15	0.65 (0.41, 1.04)	0.07	0.68 (0.43, 1.08)	0.10	0.68 (0.42, 1.08)	0.10
**Pet health status**								
Poor	1.00		1.00		1.00		1.00	
Fair	0.41 (0.24, 0.70)	< 0.01	0.41 (0.23, 0.70)	< 0.01	0.40 (0.23, 0.69)	< 0.01	0.41 (0.24, 0.71)	< 0.01
Good	0.59 (0.34, 1.04)	0.06	0.58 (0.33, 1.02)	0.06	0.57 (0.32, 1.00)	0.05	0.61 (0.34, 1.07)	0.09
**Annual pet-related expenses (RMB yuan)**								
< 1000	1.00		1.00		1.00		1.00	
1000–2999	0.72 (0.44, 1.19)	0.21	0.75 (0.47, 1.24)	0.26	0.71 (0.43, 1.17)	0.18	0.71 (0.43, 1.18)	0.19
≥3000	0.45 (0.20, 1.03)	0.06	0.48 (0.21, 1.12)	0.09	0.45 (0.19, 1.02)	0.06	0.44 (0.19, 1.02)	0.06
**Pet walking frequency**								
Almost every day	1.00		1.00		1.00		1.00	
Several times a week	1.75 (1.01, 3.04)	< 0.05	1.92 (1.11, 3.32)	< 0.05	1.79 (1.03, 3.11)	< 0.05	1.79 (1.04, 3.10)	< 0.05
Rarely	1.58 (0.88, 2.84)	0.12	2.04 (1.17, 3.55)	< 0.05	1.97 (1.13, 3.42)	< 0.05	1.87 (1.08, 3.25)	< 0.05
**Daily interaction time with pets**								
< 1 h	1.00		1.00		1.00		1.00	
1–2 h	1.74 (0.79, 2.26)	0.27	1.34 (0.79, 2.27)	0.27	1.29 (0.76, 2.18)	0.34	1.27 (0.75, 2.15)	0.37
>2 h	2.16 (1.22, 3.80)	< 0.01	2.26 (1.27, 4.00)	< 0.01	2.25 (1.27, 3.99)	< 0.01	2.18 (1.24, 3.84)	< 0.01
Dog ownership							1.00	
No	1.00							
Yes	0.63 (0.40, 0.98)	< 0.05						
**Cat ownership**								
No			1.00					
Yes			0.82 (0.55, 1.23)	0.34				
**Bird ownership**								
No					1.00			
Yes					1.87 (0.96, 3.62)	0.06		
**Rabbit ownership**								
No							1.00	
Yes							1.94 (1.04, 3.59)	< 0.05

All statistical analysis included sociodemographic characteristics.

## 4 Discussion

This study, based on large-sample, nationwide cross-sectional data, provides the first systematic assessment of the association between pet ownership and sleep quality among Chinese older adults. Our findings indicate that pet ownership is significantly associated with a lower risk of insomnia (OR = 0.75), consistent with prior evidence from Western populations suggesting beneficial effects of companion animals on psychological well-being and sleep regulation (20). Further analysis indicates that this association is influenced by multiple factors, including the duration of pet ownership, the pet's health status, and pet type—findings that require in-depth mechanistic interpretation and consideration of the unique context of older adults in China. The association between pet ownership and better sleep quality observed in this study may reflect several intersecting psychological, behavioral, and social pathways.

The relationship between pet ownership duration and sleep quality is time-dependent. This study found that longer the duration of pet ownership was associated with a lower risk of insomnia, which may be attributed to the gradual establishment of strong emotional attachment and sustained emotional support between older adults and their pets over time (21). This attachment bond serves to mitigate loneliness and anxiety, promoting the release of oxytocin while attenuating sympathetic nervous system activation. This physiological cascade fosters a state of relaxation, thereby facilitating both sleep onset and maintenance (10, 12). Conversely, during the short-term pet ownership phase (less than 1 year), older adults may experience sleep disruption as they adapt to pet care demands. Fundamentally, this may stem from the disruption of homeostasis. Older adults typically maintain entrenched circadian rhythms and lifestyle habits; the introduction of a new pet necessitates significant behavioral adjustments, such as altering wake-up times or managing nocturnal disturbances. This perturbation of the established daily order can trigger “acute caregiver stress,” leading to a transient elevation in cortisol levels that impairs sleep initiation (11, 21). We observed that older adults engaging in over two hours of daily interaction with their pets had a higher risk of insomnia. This association may be explained by the fact that prolonged interaction duration could serve as a proxy for underlying challenges, such as behavioral issues in pets (e.g., separation anxiety) that may also cause sleep disruption at night, or owner-related anxiety and over-attachment that sustains a state of hypervigilance (12). Furthermore, stimulating interactions close to bedtime may directly interfere with the wind-down process necessary for sleep onset.

Pet health status is closely linked to older adults' sleep quality. This study found that owners of unhealthy pets faced a significantly higher risk of insomnia (OR = 0.41 for healthy vs. unhealthy pets).

First, in the specific socioeconomic context of China, pet illness frequently entails severe “financial toxicity” (22). Unlike Western nations, China possesses a nascent pet medical insurance system with negligible coverage, whereas veterinary costs remain relatively high (23). For older adults who primarily rely on fixed pensions, sudden veterinary expenditures can impose a substantial economic shock. This financial vulnerability often triggers intense anxiety and leads to nocturnal “rumination”, characterized by repetitive dwelling on medical bills and the prognosis of the pet (24). Such cognitive activity significantly delays sleep initiation. Second, the physical discomfort of an unwell pet often causes nocturnal restlessness that directly fragments the owner's sleep (13, 25). Beyond these direct interruptions, chronic caregiving responsibilities can induce a persistent state of “hyperarousal”. This heightened vigilance activates the hypothalamic-pituitary-adrenal (HPA) axis and leads to sustained elevation of cortisol levels. Consequently, this physiological response disrupts sleep homeostasis, trapping older adults in a vicious cycle of physical exhaustion accompanied by an inability to fall asleep (21).

Significant differences in sleep effects were observed across pet types. We found that dog ownership was linked to a lower risk of insomnia, whereas rabbit ownership was associated with a higher risk. No significant associations were observed for cat or bird ownership. These disparities likely arise from distinct interaction patterns, care demands, and associated psychosocial impacts. The beneficial effect of dog ownership may be attributed to several mechanisms. Dog owners typically engage in more physical activity (averaging approximately 45 min of daily walking in this study), which helps regulate circadian rhythms and improve sleep efficiency (26, 27). Walking dogs provides more social opportunities, reducing loneliness and indirectly promoting sleep (28). Moreover, dog owners experience greater daytime exposure to natural light, which helps maintain healthy circadian rhythms (29). These mechanisms have been validated across multiple countries and diverse populations (30–33), and our study further confirms them among Chinese older adults. Conversely, rabbit ownership was associated with an increased risk of insomnia. Rabbits are predominantly nocturnal animals requiring cage confinement and demanding care (e.g., frequent cage cleaning), which may increase owners' psychological stress and correlate with poorer sleep quality (34). As for cat and bird ownership, the lack of significant association with insomnia risk may reflect a balance of counteracting factors. For cats, calming interactions such as petting can provide emotional support and reduce stress, potentially benefiting sleep (28). However, cats are often most active during dawn and dusk, and their nighttime behaviors can disrupt an owner's sleep, particularly when co-sleeping occurs (33). Similarly, bird ownership shows no significant association with sleep quality. The relatively low intensity of human-bird interaction and lighter caregiving burden. Although the visual and auditory stimulation provided by birds may offer psychological benefits and mild stress relief (35), these advantages do not necessarily translate into measurable improvements in sleep quality.

Sociodemographic factors play a moderating role in the association between pet ownership and sleep. Regarding marital status, married individuals can share the emotional and practical burdens of pet care with a spouse (36), thus promoting better sleep. Whereas single pet owners, especially when faced with a pet's health issues, may shoulder the full burden alone, increasing their risk of loneliness and sleep problems (12, 37). Educational attainment also appears to be a key moderator, as higher education is often associated with greater health literacy, superior stress management skills, and stronger economic resilience—all of which are conducive to maintaining good sleep (38). Residential setting played a role in our study, with rural elderly reporting better sleep quality than urban dwellers. This may be attributed to the quieter environment and superior air quality in rural areas, alongside a lifestyle that typically involves more physical labor and outdoor activity (39). Although urban areas offer more developed infrastructure (40), these benefits appear to be offset by detrimental factors such as a faster pace of life, noise, light pollution, and poorer air quality, which can collectively disrupt sleep (41).

This study has several limitations. First, the cross-sectional design prevents definitive causal inferences regarding the relationship between pet ownership and sleep quality. Longitudinal studies are needed to clarify temporal patterns and address the possibility of reverse causality. Second, participants were recruited from community health service centers rather than through population-based random sampling, which may restrict the generalizability of the findings to the broader older adult population in China. Future studies should validate these findings through community-based random sampling across broader geographic regions. Third, sleep quality assessment relied on self-reported questionnaires. Although the AIS is a validated and widely used instrument for detecting insomnia symptoms, self-reported measures may be subject to recall or reporting bias. Objective sleep indicators, such as sleep duration, sleep efficiency, and nighttime awakenings, were not captured in this study. Future research incorporating subjective and objective assessments would provide a more comprehensive evaluation of sleep health. Fourth, although the analysis adjusted for multiple sociodemographic and lifestyle factors, important psychosocial and environmental variables-such as loneliness, social support, chronic disease severity, sleep environment, and co-sleeping with pets-were not assessed. These unmeasured factors may contribute to residual confounding, and future studies should incorporate more comprehensive measures to clarify the pathways linking pet ownership with sleep quality in older adults.

Based on these findings, we propose several actionable strategies to optimize sleep health for older pet owners. Dog owners may benefit from maintaining regular daytime routines, such as scheduled walking and consistent interaction, which help reinforce circadian rhythm and increase physical activity. Older adults who are new to pet ownership may require brief guidance on establishing predictable feeding and activity schedules to minimize nighttime disruptions during the adjustment period. In addition, owners of pets with poorer health may experience greater caregiving stress; providing accessible veterinary support and simple advice on managing pet-related responsibilities may help reduce sleep disturbance. These focused strategies highlight practical ways in which pet companionship can be optimized to support, rather than disrupt, sleep in later life.

## 5 Conclusions

This study suggests that pet ownership may serve as a potential intervention to promote sleep health among older adults, with its effectiveness influenced by the duration of pet ownership, the pet's health status, and the type of pet. Future research employing longitudinal designs and intervention trials could further explore the mechanisms underlying pet ownership's role in healthy aging, thereby providing higher-level evidence to support the development of “pet-friendly” environments conducive to healthy aging.

## Data Availability

The raw data supporting the conclusions of this article will be made available by the authors, without undue reservation.
